# Therapeutic Notes

**Published:** 1898-12

**Authors:** 


### THERAPEUTIC NOTES.

UNDER THE DIRECTION OF W. J. MARTIN, M.D.C.,
KANKAKEE, ILL.

BARIUM CHLORIDE.—At the last meeting of the Illinois State Veterinary Society the treatment of the various forms of equine colic was thoroughly discussed. While there was a wide diversity of opinion among the members concerning the efficacy of the various drugs used in combating this disease, it seemed to be the opinion of nearly all those present that barium chloride, administered either by the mouth or intravenously, was a dangerous drug, one likely to produce serious systemic derangement if not actually fatal results.

SUCCUS CINERARIA MARITIMA FOR CATARACT.—Cineraria maritima is a plant indigenous to the Island of Trinidad, West Indies. The juice of this plant is again being used in the treatment of cataract in human practice. Judging from the favorable reports in current medical literature, this remedy is having astonishing success in removing cataract. According to the latest information concerning this drug's action, it is said that the juice does not appear to produce any irritability or inflammation; a slight burning sensation lasting for about two minutes, but which gradually passes away, follows its application. There is also more or less profuse lachrymal discharge, tinged with the coloring-matter of the liquid used.

The action of the drug consists in stimulation of the absorbents, a dissolution of the opaque matter in the crystalline lens or its capsule ensuing. As far as present experience has gone, the improvement is progressive and enduring. Two drops are instilled into the eye twice daily. The price of the drug is one dollar per drachm vial. The remedy might be used in veterinary practice with equally good results.

A MIXTURE consisting of equal parts of gum-camphor and carbolic acid crystals is of value in treating the troublesome "summer" or "bursatee" sores so common on the legs and feet of horses and mules during the summer months. The mixture is best applied with a small brush several times a day, the sores afterward covered with cotton-wool, and a bandage applied.

Saline Solution for Burns.—Tomasoli, quoted in *Medical Record* of Janurary 29, 1898, recommends the intravenous injection of a solution of sodium chloride and sodium bicarbonate to combat the fatal effects of extensive burns. In a series of experiments carried out by Tomasoli with ten dogs which were scalded by immersion in boiling water, but two of the animals died that had received the serum injection.

Artificial Blood-serum for Subcutaneous or Intravenous Injections.—℞. Calcium chloride, grs. iv; potassium chloride, grs. xv; sodium chloride, ʒij et grs. xv; boiled or distilled water, ℥xxxv. M.

New Chemical Orthography.—The following changes in chemical spelling have been adopted by the Chemical Section of the American Association for the Advancement of Science: 1. Drop final *e* in names of chemicals ending in *ide*, and pronounce *id*, as, for instance, "chlorid," "bromid," "iodid." This avoids confusion with the terminal *ite*, as in "chlorite." Also write and pronounce carbid, hydrid, amilid, glycerid, etc.

2. Drop final *e* in names of elements and compounds ending in *ine*, writing chlorin for chlorine, glycerin for glycerine, antipyrin for antipyrine, codein for codeine, etc. This rule also embraces the alkaloids, which ought to be written without a final *e*.

3. Use the termination *ol* without *e*, and pronounce short in the case of specific chemical compounds, exclusively for alcohols, such as phenol, creosol, glycol, glycerol. The termination *ole* is pronounced long and is limited to preparations that are not alcohols, as indol; also in galenical preparations, like glycerole of starch.

4. In sulphur and all derivatives therefrom substitute "*f*" for "*ph*," as in *sulfur*. Many of the leading drug and chemical journals in the country have adopted the above method of writing and spelling chemical names.

Mayol.—E. May, of Budapest, has introduced a preservative under the above name, which, according to Thaus (*Pharm. Centralbl.*) consists of ethyl and methyl alcohols, boric acid, ammonium, fluorid and glycerin. Meat painted with this solution will remain fresh for weeks, becoming coated with a dry crust.

A New Remedy for Tuberculosis.—At the meeting of the American Medical Association held recently in Denver, Dr.

J. B. Murphy, of Chicago, read a remarkable paper on a new method lately discovered by him for the cure of tuberculosis. Dr. Murphy's discovery is one of great simplicity, and, on this account, can be readily used by the private practitioner in his daily practice.

Dr. Murphy claims, and with every probability of truth, that by placing a lung affected with tuberculosis in a state of collapse (state of rest) cicatrization will take place around the diseased area of the lung. The method of performing this consists in injecting into the pleural cavity nitrogen gas under hydraulic pressure. This injection of nitrogen gas into the pleural cavity relieves the lung from the performance of all physiological activity which has a tendency to diminish the irritating cough and accompanying expectoration, which are so wearing upon the vital forces of a tubercular patient. It is to this freedom from cough that Dr. Murphy attributes the curative method of his treatment. After the period of rest, and when cicatrization of the diseased areas of the lung have taken place, the lung can again be placed in a state of physiological activity by simply removing by aspiration with a trocar the nitrogen gas from the lung. Dr. Murphy claims that in five cases of advanced tuberculosis treated by this method all were cured.

This method of treating tuberculosis is, at the present time, being given an extensive trial in the Chicago hospitals, and it remains to be seen whether this innovation will stand the crucial test or be destined to add another disappointment to the many that have endeavored to find a cure for this dread scourge of the human species.

Active Constituents of Digitalis.—Digitalis contains four active physiological principles, namely, digitalin, digitoxin, digitalein, and digitonin. The effect of digitalin is that of a powerful cardiac stimulant, increasing the flow of blood and also acting as a stimulant to the vasomotor centres. The action of digitalein and digitoxin are the same as digitalin, with the exception that they do not stimulate the vasomotor centres.

Painless Arsenical Paste.—As an escharotic to malignant growths, such as epithelial cancer, fungoid growths, etc., the following paste may be used with decided benefit: ℞.—Arsenic, g. 1; orthoform, g. 1; alcohol, g. 75; water, g. 75. M. The strength of this preparation may be increased or decreased by increasing or decreasing the amount of the water and alcohol.

Hydroquinine.—Hydroquinine, which is quinine containing an additional hydroxyl radical, is a virulent poison, producing convulsions and death in small doses. It is produced by treating an alcoholic solution of quinine with metallic sodium.

Granulated Opium for Making Tincture of Opium.—Granulated opium is the best form of this drug to use in preparing tincture of opium. A tincture from this form of the drug was found to contain a higher per cent. of morphine (1.433) than from the various other forms of the drug used.

Corrosion of Surgical Instruments.—Surgical instruments are not corroded by corrosive sublimate solution, providing the solution has been first rendered alkaline by the addition of borax, sodium bicarbonate, or sodium benzoate.

Commercial Plants of Europe.—About 4200 different species of plants are gathered and used in Europe for commercial and medicinal purposes.

Pyramidon.—This is a new antipyretic and analgesic, and, to a certain extent, may be used as a substitute for antipyrine, from which it is a derivative. It is a yellowish-white, crystalline powder, soluble in water in the proportion of 1 to 10, and is practically tasteless. The physiological action of pyramidon is similar to antipyrine, and the dose is about the same as of that drug, although a more lasting effect is claimed for pyramidon than for antipyrine.

Tetanus Antitoxin.—The use of this serum seems to be on the increase in human practice. Several cases of acute tetanus have been lately reported in the medical journals as being cured by its use. On the other hand, in veterinary practice the general consensus of opinion seems to be that this serum is of but little benefit either as a prophylactic or a curative agent.

Antistreptococcic Serum.—A case of puerperal fever in a woman has recently been cured by the use of this serum.

Medical Men as Morphine Eaters.—In 1000 cases of chronic morphine eaters, medical men formed 40 per cent. of the number.

